# Pentose Phosphate Shunt Modulates Reactive Oxygen Species and Nitric Oxide Production Controlling *Trypanosoma cruzi* in Macrophages

**DOI:** 10.3389/fimmu.2018.00202

**Published:** 2018-02-16

**Authors:** Sue-jie Koo, Bartosz Szczesny, Xianxiu Wan, Nagireddy Putluri, Nisha Jain Garg

**Affiliations:** ^1^Department of Pathology, University of Texas Medical Branch (UTMB), Galveston, TX, United States; ^2^Department of Anesthesiology, University of Texas Medical Branch (UTMB), Galveston, TX, United States; ^3^Department of Microbiology and Immunology, University of Texas Medical Branch (UTMB), Galveston, TX, United States; ^4^Department of Molecular and Cell Biology, Baylor College of Medicine, Houston, TX, United States; ^5^Institute for Human Infections and Immunity, University of Texas Medical Branch (UTMB), Galveston, TX, United States

**Keywords:** metabolism, macrophages, reactive oxygen species, peroxisome proliferator-activated receptors, NADPH, *Trypanosoma cruzi*, pentose phosphate pathway

## Abstract

Metabolism provides substrates for reactive oxygen species (ROS) and nitric oxide (NO) generation, which are a part of the macrophage (Mφ) anti-microbial response. Mφs infected with *Trypanosoma cruzi* (*Tc*) produce insufficient levels of oxidative species and lower levels of glycolysis compared to classical Mφs. How Mφs fail to elicit a potent ROS/NO response during infection and its link to glycolysis is unknown. Herein, we evaluated for ROS, NO, and cytokine production in the presence of metabolic modulators of glycolysis and the Krebs cycle. Metabolic status was analyzed by Seahorse Flux Analyzer and mass spectrometry and validated by RNAi. *Tc* infection of RAW264.7 or bone marrow-derived Mφs elicited a substantial increase in peroxisome proliferator-activated receptor (PPAR)-α expression and pro-inflammatory cytokine release, and moderate levels of ROS/NO by 18 h. Interferon (IFN)-γ addition enhanced the *Tc*-induced ROS/NO release and shut down mitochondrial respiration to the levels noted in classical Mφs. Inhibition of PPAR-α attenuated the ROS/NO response and was insufficient for complete metabolic shift. Deprivation of glucose and inhibition of pyruvate transport showed that Krebs cycle and glycolysis support ROS/NO generation in *Tc* + IFN-γ stimulated Mφs. Metabolic profiling and RNAi studies showed that glycolysis-pentose phosphate pathway (PPP) at 6-phosphogluconate dehydrogenase was essential for ROS/NO response and control of parasite replication in Mφ. We conclude that IFN-γ, but not inhibition of PPAR-α, supports metabolic upregulation of glycolytic-PPP for eliciting potent ROS/NO response in *Tc-*infected Mφs. Chemical analogs enhancing the glucose-PPP will be beneficial in controlling *Tc* replication and dissemination by Mφs.

## Introduction

*Trypanosoma cruzi* (*T. cruzi* or *Tc*) is a blood-borne, protozoan parasite that causes Chagas cardiomyopathy and is endemic in Latin American countries. The parasite has broad host and tissue tropism. Two anti-parasite drugs, benznidazole and nifurtimox, are used in the early phase of infection, yet these drugs require long-term treatment and cause toxic side effects (1).

Macrophages (Mφs) are one of the first responders to infection. Toll-like receptors (TLRs) expressed by Mφs and other innate immune cells recognize the pathogen associated molecular patterns and transmit a signal *via* cytoplasmic Toll/interleukin (IL)-1R domains. Subsequently, cytosolic adaptor molecules, including myeloid differentiation primary-response protein 88 (MyD88), are recruited and induce nuclear factor-κB (NF-κB) activation, leading to the production of inflammatory cytokines and linking the innate to the adaptive immune responses (2, 3). Indeed, *Tc*-derived glycosylphosphatidylinositols (GPIs) and GPI-anchored mucin-like glycoproteins are shown to engage TLR2, TLR4, and TLR9 to stimulate the synthesis of IL-12 and TNF-α by Mφs (4). Further, interaction of *Tc* with Mφs induces a substantial increase in the expression and secretion of pro-inflammatory cytokines at a level similar to that seen in classically activated pro-inflammatory Mφs stimulated by lipopolysaccharide (LPS) + IFN-γ treatment (5). Yet, Mφs are not able to kill *Tc*, especially the virulent isolates of the parasite (6).

Besides cytokines and chemokines, activated Mφs exert cytotoxic effects against microbes by production of reactive oxygen species (ROS). NADPH oxidase (NOX2), a multi-meric complex, utilizes NADPH as substrate and reduces molecular oxygen to produce superoxide (${\text{O}}_{2}^{-}$) that is then further dismutated into stable and diffusible hydrogen peroxide pro-oxidant. Studies using *in vitro* assay systems or animal models have shown that NOX2-dependent ${\text{O}}_{2}^{-}$ formation is required for parasite control in Mφs and splenocytes (7, 8). NOX2/ROS also signal the development of antigen-specific CD8^+^ T cell response required for control of tissue parasites in infected mice (9). Likewise, inducible nitric oxide synthase (iNOS) is activated by immunological stimuli in a Ca^+2^-independent manner, and utilizes l-arginine and molecular oxygen for the synthesis of l-citrulline and nitric oxide (NO) in a complex oxidoreductase reaction (10). The reaction of NO with ${\text{O}}_{2}^{-}$ produces peroxynitrite that is a strong cytotoxic oxidant shown to promote killing of *Tc* in Mφs (11, 12). However, the extent of NOX2/ROS and iNOS/NO response in human and mouse Mφs infected with *Tc* is substantially lower than that observed in LPS + IFN-γ induced, classically activated Mφs (5), thus suggesting a potential mechanism for survival of parasite in Mφs. How parasites manipulate NOX2 and iNOS activation is not fully understood.

Metabolism of Mφs is associated with differential inflammatory activation. It is suggested that pyruvate oxidation to acetyl CoA to feed the mitochondrial Krebs cycle supports the oxidative phosphorylation (OXPHOS) in anti-inflammatory and resting Mφs; and the generation of pro-inflammatory molecules is reliant on Warburg glycolysis, where the end product, pyruvate, is reduced to lactate (13). Glycolysis inhibition during LPS-induced sepsis or granulocyte macrophage colony stimulating factor and LPS stimulation have shown reduction of pro-inflammatory cytokine responses in Mφs (14, 15), while inhibition during Mφ differentiation reduces the IL-6 levels in response to LPS (16). Whether and how *Tc* prevents metabolic shift for pro-inflammatory activation of Mφs is not known.

Peroxisome proliferator-activated receptors (PPARs) are transcriptional regulators of fatty acid β-oxidation and cell proliferation. PPARs (α, β, and γ isoforms) can be activated by a variety of endogenous, natural ligands including fatty acids, and act in concert with retinoid X receptors to regulate the gene expression [(17, 18) and references therein]. PPAR-γ has been intensively studied as a regulator of adipogenesis and also suggested to play an immuno-regulatory function in Mφs (19). The α and δ isotypes of PPAR have been implicated in the control of fatty acid oxidation in the skeletal muscle, liver, and heart, and are comparatively less studied in innate immune cells. An experimental model of Chagas disease has shown that at 6 days post-infection, Mφs isolated from the peritoneum demonstrate increase in PPAR-α, PPAR-γ, and iNOS mRNA (20). These isolated Mφs responded to PPAR-α and -γ agonists by declining NO levels and mRNA of pro-inflammatory cytokines, and increase in parasite uptake, thus suggesting a potential role of PPARs in regulating the immune cell response to *Tc* infection.

In this study, we investigated if a metabolic shift is essential for pro-inflammatory function of Mφs, and whether *Tc* induces PPAR-dependent metabolic perturbations that result in poor activation of Mφs. For this, we utilized primary and cultured wild-type (WT) and PPAR-α^−/−^ Mφs and small molecule agonists and antagonists of PPARs and metabolic pathways, and employed biochemical techniques, Seahorse Extracellular Flux Analyzer, and liquid chromatography-mass spectrometry. The NOX2 and iNOS enzymes utilize molecular oxygen and NADPH as substrate. We, therefore, also examined how metabolic pathways, which produce NADPH, contribute to limited NOX2 and iNOS activation in infected Mφs, and employed an RNAi approach to identify the rate-limiting step that is essential for macrophage cytotoxic response against *Tc*.

## Results

### NOX2-Dependency of ROS Production in *Tc* + IFN-γ Stimulated Mφs

**Figure 1:**
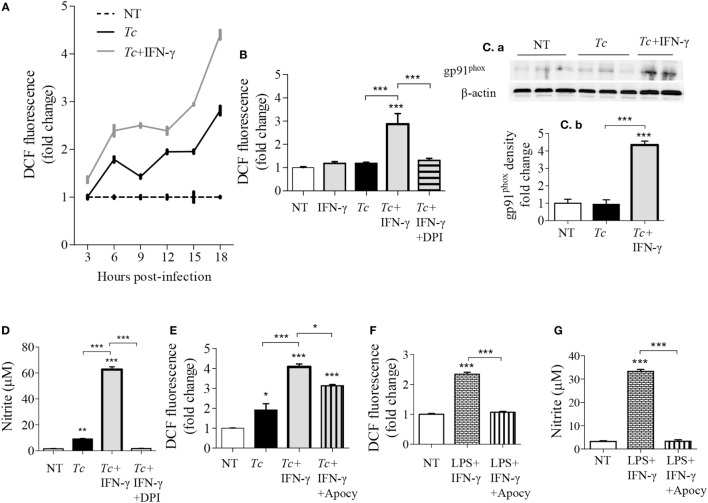
NOX2-dependency of reactive oxygen species (ROS)/nitric oxide (NO) generation in macrophages stimulated with *Trypanosoma cruzi* (*Tc)*. RAW264.7 macrophages were stimulated with *Tc* for 18 h in presence or absence of IFN-γ and inhibitors of NADPH oxidase, diphenyliodinium (DPI), or apocynin (apocy). **(A)** Intracellular ROS levels were measured for 3–18 h post-infection (pi) by DCF fluorescence. **(B,E)** ROS levels with NOX2 inhibition by DPI **(B)** and apocynin **(E)**. **(C)** Immunoblot analysis of gp91phox in macrophage cell lysates at 18 h pi [**(C)**a], normalized to β-actin loading control [**(C)**b]. **(D)** Nitrite release by infected macrophages at 18 h, with NOX2 inhibition by DPI, was measured by Griess assay. **(F,G)** Macrophages activated with lipopolysaccharide + IFN-γ in presence or absence of apocynin for 18 h. The ROS level **(F)** and NO release **(G)** were analyzed as above. All data are shown as mean ± SEM of ≥3 biological replicates (triplicate observations per replicate). Statistical significance (ANOVA_Tukey_) is presented by *p < 0.05, **p < 0.01, and ***p < 0.001 compared to no-treatment controls unless marked by a line over comparing groups.

We have previously observed low intracellular ROS levels in *Tc-*infected Mφs, but a significant increase of ROS in LPS + IFN-γ stimulated Mφs (5). We, therefore, first monitored if IFN-γ amplifies the mφ response to *Tc*. RAW264.7 Mφs responded to *Tc* with an increase in ROS level at 3 h that continued through 18 h post-infection (pi) (Figure 1A). IFN-γ alone did not elicit ROS production; however, co-incubation with IFN-γ enhanced the *Tc*-induced ROS level by 2.5-fold in infected Mφs (Figures 1A,B, *p* < 0.001). The high increase in ROS content by 18 h was directly associated with an increase in the protein levels of the gp91phox, the catalytic subunit of NADPH oxidase (NOX2, Figures 1Ca,b, *p* < 0.001), and ROS response declined by the NOX2 inhibitors, diphenyliodinium (DPI) (Figure 1B, *p* < 0.001) and apocynin (Figure 1E, *p* < 0.001) in *Tc* + IFN-γ stimulated Mφs. NO response was also abolished by DPI treatment in *Tc* + IFN-γ stimulated Mφs (Figure 1D, *p* < 0.001). Likewise, apocynin treatment abolished the LPS + IFN-γ induced ROS production (Figure 1F, *p* < 0.001) and NO release (Figure 1G, *p* < 0.001) in Mφs. These results indicate that the ROS production in Mφs responding to infection in the presence of IFN-γ is NOX2-dependent, and correlates with the protein abundance of NOX2 catalytic unit.

### Expression of PPAR Isoforms and Effect of PPAR-α Modulation on Cytokine Response in Mφs Infected with *Tc*

**Figure 2:**
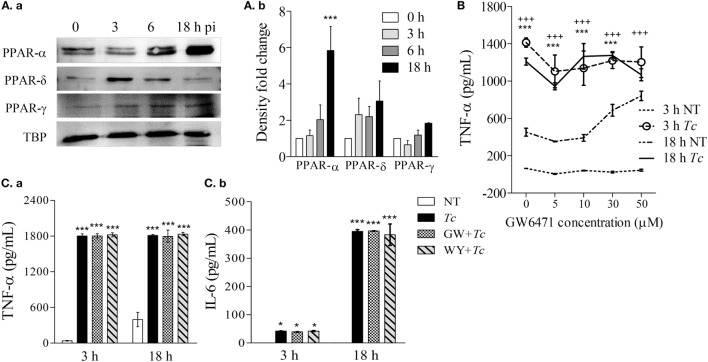
Expression of peroxisome proliferator-activated receptor (PPAR) isoforms and effect of PPAR-α modulation on cytokine response in macrophages infected by *Trypanosoma Cruzi* (*Tc*). RAW 264.7 macrophages were infected with *Tc* for 0, 3, 6, and 18 h. **(A)** Nuclear abundance of PPAR isoforms and TATA binding protein (TBP, loading control) in infected cells was determined by immunoblotting [**(A)**a]. Densitometry calculation of PPAR signal was performed in three experimental replicates, and normalized to TBP [**(A)**b]. **(B)** Titration of PPAR-α inhibitor concentration. Macrophages were incubated for 3 or 18 h with *Tc* (±GW6471, PPAR-α inhibitor; 0–50 µM). Supernatants were analyzed for TNF-α levels by an enzyme linked immunosorbant assay (ELISA). **(C)** PPAR-α role in cytokine release. Macrophages were incubated with *Tc* in presence or absence of 10 µM GW6471 (GW) or 50 µM WY14643 (WY, PPAR-α agonist) for 3 or 18 h. TNF-α [**(C)**a] and interleukin (IL)-6 [**(C)**b] levels in supernatants were determined by an ELISA. Data in bar graphs are shown as mean value ± SEM of ≥3 biological replicates (triplicate observations per replicate). ANOVA_BF_ *p < 0.05 and ***p < 0.001 compared to time point 0 [**(A)**b], no-treatment controls **(C)**, or no-treatment controls at 18 h **(B)**. ^+++^*p* < 0.001 compared to no-treatment controls at 3 h **(B)**.

All of the three PPAR isoforms are prime regulators of lipid metabolism in various cell types and may control mφ metabolism. In order to determine whether PPARs are involved in mφ response to *Tc*, we first evaluated the nuclear expression of PPAR isoforms in RAW264.7 Mφs infected with *Tc* for 0, 3, 6, and 18 h. These data showed that the nuclear expression of PPAR-α was increased by fivefold at 18 h pi (Figures 2Aa,b, *p* < 0.001). The nuclear abundance of δ and γ isoforms of PPARs did not change significantly over the course of *Tc* infection (Figures 2Aa,b). To test the role of PPAR-α in modulating pro-inflammatory response, we co-incubated the Mφs with *Tc* and small molecule inhibitor of PPAR-α (GW6471, 5–50 µM). RAW264.7 Mφs incubated with *Tc* for 3 or 18 h exhibited 2- to 22-fold increase in TNF-α release that was not heightened or diminished upon co-incubation with increasing concentrations of GW6471 (Figures 2B,Ca). Co-incubation of Mφs with PPAR-α small molecule agonist (WY14643, 50 µM) also did not dampen the TNF-α release at 18 h pi (Figure 2Ca). Likewise, Mφs infected with *Tc* for 18 h exhibited >300-fold increase in IL-6, and no effects of PPAR-α agonist and antagonist were observed on *Tc*-induced IL-6 release in Mφs (Figure 2Cb). Higher concentrations of GW6471 (30–50 µM) induced TNF-α release in normal Mφs (Figure 2B), which suggested a toxic effect, and therefore were not used in subsequent experiments. Together, these results suggest that *Tc* infection induces a significant increase in PPAR-α expression and TNF-α and IL-6 production in Mφs; however, PPAR-α is not the prime regulator of the pro-inflammatory cytokine response in infected Mφs.

### PPAR-α Modulation of ROS and NO Response in Mφs Infected with *Tc*

**Figure 3:**
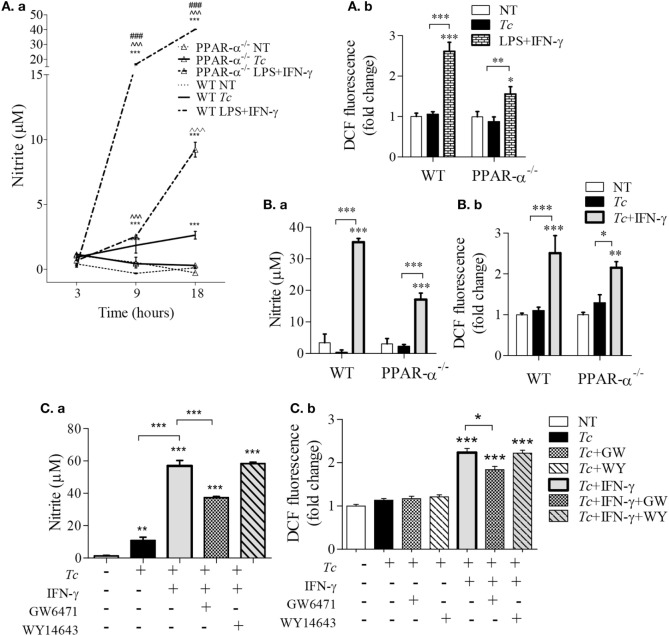
Peroxisome proliferator-activated receptor (PPAR-α) modulation of reactive oxygen species (ROS) and nitric oxide (NO) response in macrophages [±*Trypanosoma cruzi* (*Tc*)]. **(A,B)** Bone marrow-derived monocytes of wild-type (WT) and PPAR-α^−/−^ mice were matured into macrophages, and incubated with *Tc, Tc* + IFN-γ, or lipopolysaccharide (LPS) + IFN-γ for 3, 9, and 18 h. The time course of NO release [**(A)**a] and NO release at 18 h [**(B)**a] were determined by Griess assay. The intracellular ROS levels were measured by DCF fluorescence [**(A)**b and **(B)**b]. **(C)** RAW264.7 macrophages were incubated with *Tc* at the same time in presence or absence of IFN-γ, GW6471 (PPAR-α antagonist), or WY14643 (PPAR-α agonist) for 18 h. The NO release [**(C)**a] and intracellular ROS [**(C)**b] were measured as above. Data are shown as mean ± SEM of ≥3 biological replicates with triplicate observations per replicate. Significance was calculated by ANOVA_Tukey_ or ANOVA_BF_, and shown as **p < 0.01, ***p < 0.001 compared to no-treatment controls unless marked by line over comparing groups; ^^^^^*p* < 0.001 compared to infection only group; ^###^*p* < 0.001 compared to PPAR-α^−/−^ LPS + IFN-γ treatment.

As NO and ROS are important antimicrobials, we examined if PPAR-α is a regulator of these compounds in infected Mφs. We first used bone marrow-derived Mφs (BMDMs) from WT and PPAR-α^−/−^ mice. LPS + IFN-γ treatment of WT BMDMs increased the nitrite levels, the breakdown product of NO, from 1 to 16 µM within 9 h and to 40 µM in 18 h (Figure 3Aa, *p* < 0.001). The PPAR-α^−/−^ BMDMs also exhibited a significant increase in nitrite in response to LPS + IFN-γ; however, this response was fourfold lower than that noted in WT BMDMs (Figure 3Aa, *p* < 0.001). Similarly, WT and PPAR-α^−/−^ BMDMs exhibited 2.5-fold and 50% increase in intracellular ROS levels, respectively, in response to LPS + IFN-γ treatment (Figure 3Ab, all, *p* < 0.05). When infected with *Tc*, both WT and PPAR-α^−/−^ BMDMs produced low levels of nitrite (<5 μM) by 18 h pi (Figure 3Aa). No increase in DCF fluorescence was observed in WT and PPAR-α^−/−^ BMDMs in response to *Tc* infection for 18 h (Figure 3Ab). Addition of IFN-γ resulted in >5-fold increase in NO release (Figure 3Ba, *p* < 0.001) and >1.6-fold increase in intracellular ROS level (Figure 3Bb, *p* < 0.05) in *Tc*-infected WT and PPAR-α^−/−^ BMDMs, the higher levels being observed in WT BMDMs. Likewise, RAW264.7 Mφs responded to *Tc* infection with an increase in nitrite release that was enhanced by threefold when macrophages were co-incubated with *Tc* and IFN-γ (Figure 3Ca, *p* < 0.001). PPAR-α antagonist (GW6471; 10 µM) resulted in a partial suppression (Figures 3Ca,b, *p* < 0.05), while PPAR-α agonist (WY14643; 50 µM) had no effect on *Tc* + IFN-γ induced ROS and NO levels in RAW264.7 Mφs at 18 h. Increasing concentrations of WY14643 treatment did not significantly change the NO (Figure S1 in Supplementary Material) or TNF-α (data not shown) levels in the absence or presence of *Tc*. Together the results presented in Figure 3 suggest that (a) *Tc* stimulates low levels of ROS/NO in primary and cultured Mφs, (b) co-incubation with IFN-γ enhances the *Tc*-induced ROS/NO response similar to the levels noted in LPS + IFN-γ stimulated Mφs. Further, (c) genetic and chemical inhibition of PPAR-α moderately diminished the ROS/NO response, thus suggesting that PPAR-α only partly contributes to ROS/NO production in Mφs infected by *Tc*. PPAR-α agonist had no effect on *Tc*-induced ROS/NO response in infected Mφs.

### PPAR-α Regulation of Metabolism in Mφs (± *Tc*)

**Figure 4:**
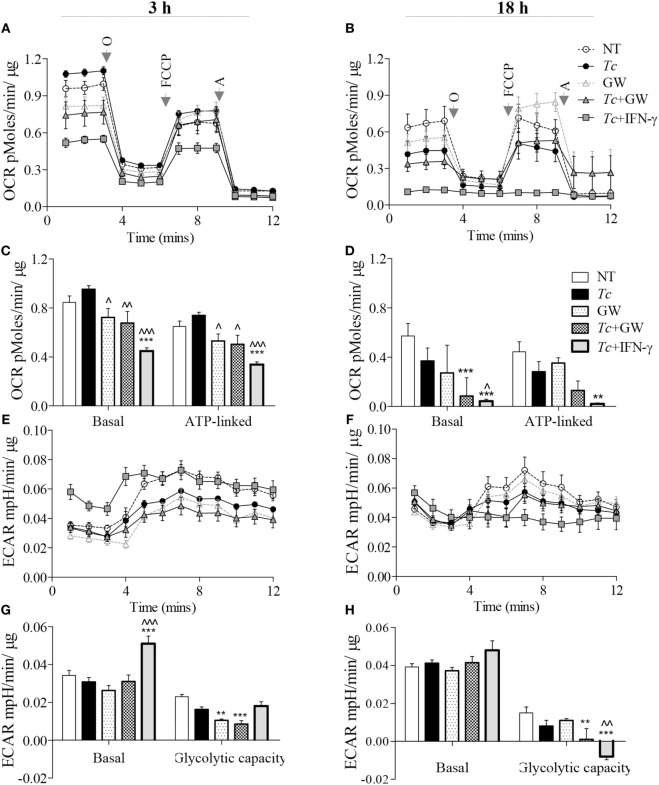
Metabolic function of macrophages infected with *Trypanosoma cruzi* (*Tc*) (±PPAR-α inhibitor). RAW 264.7 macrophages were incubated with *Tc* at the same time in presence or absence of IFN-γ and GW6471 (PPAR-α inhibitor) for 3 or 18 h. The oxygen consumption rate (OCR) and extracellular acidification rate (ECAR) were monitored by Seahorse Extracellular Flux Analyzer. Shown are the original recordings of OCR **(A,B)** and ECAR **(E,F)** with sequential addition of oligomycin (O), FCCP, and antimycin (A). Bar graphs show the quantification of mitochondrial stress parameters **(C,D)** and glycolytic capacity **(G,H)**. All data are shown as the mean ± SEM of four replicates. Statistical significance are marked as ANOVA_BF_ **p < 0.01 and ***p < 0.001 (infected and/or treated vs. no-treatment controls) or ^^^*p* < 0.05, ^^^^*p* < 0.01, ^^^^^*p* < 0.001 (*Tc* infected/treated vs. *Tc* infected).

For synthesizing ROS and NO, respectively, the NOX2 and iNOS enzymes require the NADPH substrate that can be produced through pentose phosphate pathway (PPP) and several enzymes of the mitochondrial Krebs cycle. Glycolysis provides substrates for both PPP and Krebs cycle, but only Krebs cycle feeds the OXPHOS pathway. We, therefore, first determined if glycolysis replaces the oxidative metabolism in infected Mφs, and if PPAR-α plays a role in this metabolic shift in infected Mφs. RAW264.7 Mφs infected with *Tc* alone maintained all aspects of mitochondrial respiration similar to no-treatment control at 3 h and exhibited 45% decline in oxidative metabolism at 18 h pi (Figures 4A–D). Treatment with PPAR-α inhibitor (GW6471) for 3 h resulted in a 14–28% decline in basal and ATP-linked oxygen consumption rate (OCR) in not-infected and infected Mφs (Figures 4A,C, *p* < 0.05). By 18 h, GW6471 alone (vs. no treatment) resulted in 50 and 20% decline in the basal and ATP-linked OCR, respectively, in Mφs, and further enhanced the *Tc*-induced decline in basal (*p* < 0.001) and ATP-linked OCR to 70 and 65%, respectively (Figures 4B,D). In comparison, co-incubation with IFN-γ abolished the basal and ATP-linked mitochondrial respiration in infected Mφs at 18 h (*p* < 0.01).

Concurrent to the decline in OCR, measurement of extracellular acidification rate (ECAR) provides a good indication of Warburg glycolysis. We observed no changes in ECAR values in Mφs incubated alone for 3 (Figures 4E,G) or 18 h (Figures 4F,H), or in presence of *Tc* (±GW6471) for 3 h. Treatment with GW6471 alone for 3 and 18 h resulted in 25–54% decline in glycolytic capacity (*p* < 0.01), and GW6471 treatment of infected Mφs resulted in 87% decline in the glycolytic capacity at 18 h. In comparison, IFN-γ treatment resulted in a 65% and 16% increase in basal ECAR, and 10% decline and complete depletion in glycolytic capacity in infected Mφs at 3 and 18 h post-incubation, respectively (Figures 4E–H, *p* < 0.01). Together, these results suggest that chemical inhibition of PPAR-α partially suppresses the oxidative metabolism, and likely contributes to substrate availability for NOX2 and iNOS through the use of Warburg glycolysis. However, PPAR-α inhibition did not result in oxidative to glycolytic metabolic shift as was noted with addition of IFN-γ in infected Mφs.

### Role of Mitochondrial Pyruvate in Macrophage Activation (±*Tc*)

**Figure 5:**
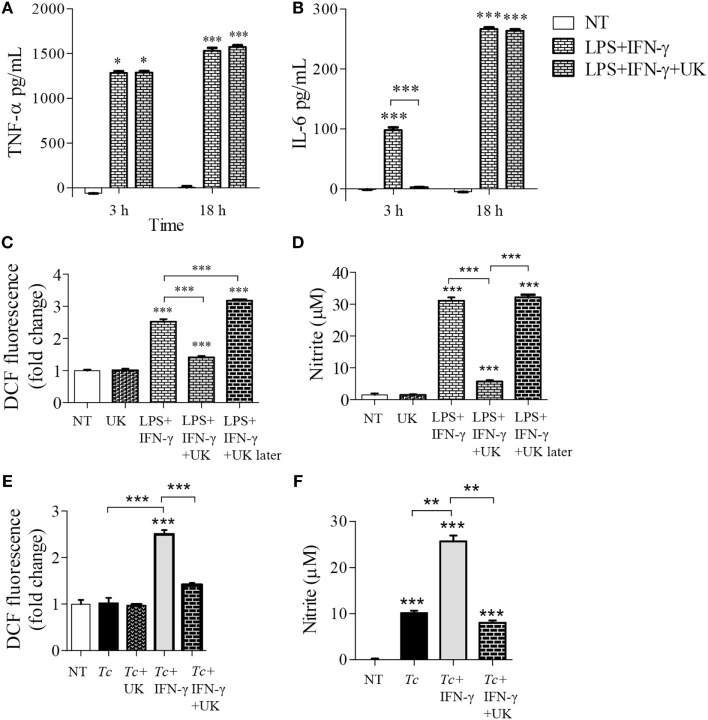
Role of mitochondrial pyruvate in the pro-inflammatory activation of macrophages. **(A–D)** RAW 264.7 macrophages were incubated with lipopolysaccharide (LPS) + IFN-γ for 3 or 18 h in the presence or absence of pyruvate transport inhibitor UK5099 (UK). **(C,D)** RAW 264.7 macrophages were sequentially incubated, first with LPS + IFN-γ for 18 h, and then with UK5099 for 4 h. **(E,F)** RAW 264.7 macrophages were incubated with *Trypanosoma cruzi* (*Tc*) at the same time in presence or absence of IFN-γ and UK5099 for 18 h. An enzyme linked immunosorbant assay was used to quantify TNF-α **(A)** and interleukin (IL)-6 **(B)** release. Intracellular reactive oxygen species **(C,E)** and nitrite release **(D,F)** were determined by DCF fluorescence and Griess assay, respectively. All data are shown as the mean ± SEM of ≥3 biological replicates (triplicate observations per replicate). Significance is presented as ANOVA_Tukey_ *p < 0.05, **p < 0.01, and ***p < 0.001 compared to no-treatment controls, and otherwise is marked by a line over comparing groups.

A broken Krebs cycle can deliver substrate for ROS/NO production as well as metabolic signal for HIF1-α-dependent pro-inflammatory activation of Mφs (21). We, therefore, further delineated the role of Krebs cycle in Mφ activation by limiting the mitochondrial availability of pyruvate (provides Acetyl CoA to feed the Krebs cycle). The Mφs stimulated for 3 or 18 h with LPS + IFN-γ produced potent quantities of TNF-α, which was not modified by co-incubation with UK5099 that inhibits pyruvate transport to mitochondria (Figure 5A). The IL-6 release was also increased in LPS + IFN-γ treated Mφs at 3 and 18 h (*p* < 0.001) which was initially delayed at 3 h with UK5099 treatment, but met the final levels of non-UK5099 treated pro-inflammatory Mφs by 18 h (Figure 5B, *p* < 0.001). Interestingly, co-incubation with UK5099 suppressed the LPS + IFN-γ induced ROS and NO levels by twofold and sixfold, respectively, in Mφs (Figures 5C,D, *p* < 0.05). Yet, UK5099 had no suppressive effect on ROS and NO levels when it was added after Mφs were stimulated with LPS + IFN-γ for 18 h (Figures 5C,D). In Mφs stimulated with *Tc* + IFN-γ, co-treatment with UK5099 suppressed the ROS and NO levels by twofold and threefold, respectively, at 18 h (Figures 5E,F, *p* < 0.01). These results suggest that mitochondrial Krebs cycle does not regulate the pro-inflammatory cytokine response, but it does support NOX2 and iNOS activities in Mφs treated with IFN-γ (+*Tc* or LPS). The pyruvate uptake-dependent Krebs cycle was dispensable in fully activated Mφs.

### Role of Glucose Uptake on Functional Activation of Mφs (± *Tc*)

**Figure 6:**
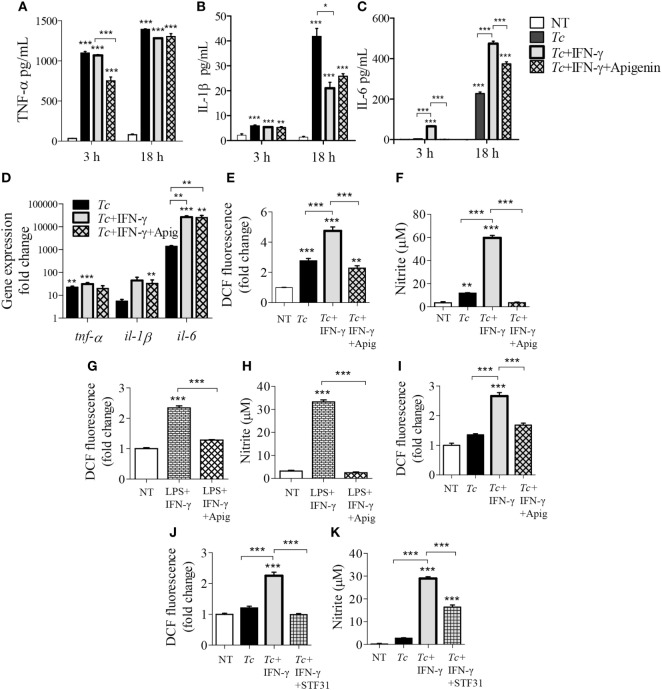
Modification of cytokine, reactive oxygen species (ROS), and nitric oxide (NO) response of activated macrophages by glucose uptake. RAW 264.7 macrophages were incubated for 3 h or 18 h with *Trypanosoma cruzi* (*Tc*) in the presence or absence of IFNγ and apigenin glucose transport 1 inhibitor **(A–C)**. Shown are quantification of TNF-α **(A)**, IL-1β **(B)**, and interleukin (IL)-6 **(C)** release by an enzyme linked immunosorbant assay and gene expression analysis of these cytokines by reverse transcription quantitative PCR **(D)**. Intracellular ROS **(E)** and nitrite release **(F)** in macrophages incubated for 18 h were determined as in Figure 5. **(G,H)** RAW 264.7 macrophages were incubated with lipopolysaccharide (LPS) + IFNγ (± apigenin) for 18 h and analyzed for ROS **(G)** and nitrite levels **(H)**. **(I)** Bone marrow-derived murine macrophages were incubated with *Tc* (±IFNγ and apigenin) for 18 h, and intracellular ROS was determined as above. **(J,K)** RAW264.7 macrophages were infected with *Tc* in the presence or absence of IFNγ and STF31 (glucose transporter 1 inhibitor) for 18 h, and intracellular ROS **(J)** and nitrite release **(K)** were measured. Data are shown as mean ± SEM of ≥3 biological replicates. ANOVA_Tukey_ *p < 0.05, **p < 0.01, ***p < 0.001 when compared to no-treatment controls unless marked by a line over comparing groups.

Expression of cytokines have been shown to be altered with the inhibition of glycolysis in pro-inflammatory macrophages (14–16). We used the glucose transporter 1 (GLUT1) inhibitor, apigenin, and observed that inhibition of GLUT1 by apigenin treatment during LPS + IFN-γ activation of macrophages result in suppressed intracellular protein (Figure S2A in Supplementary Material) and gene expression (Figure S2B in Supplementary Material) of IL-1β (*p* < 0.05) and IL-6 (*p* < 0.01) by 3 h posttreatment compared to LPS + IFN-γ treatment alone. Major changes in TNF-α levels by apigenin treatment was not observed. This suggests that apigenin treatment also reduces classically activated macrophage expression of pro-inflammatory cytokines at both the protein and gene expression levels. Next, we tested whether the expression of these inflammatory cytokines are also dependent on glycolytic shift in *Tc* + IFN-γ-activated macrophages. As noted above, *Tc* induced a significant release of pro-inflammatory cytokines (TNF-α > IL-1β > IL-6) at 3 h that was further enhanced at 18 h pi (Figures 6A–C, *p* < 0.001); and addition of IFN-γ elicited greater quantities of IL-6 in infected Mφs (Figure 6C, *p* < 0.05). The release of these cytokines did not decline greatly with the inhibition of glucose uptake during stimulation of Mφs with *Tc* + IFN-γ (Figures 6A–C). Similar observations were made at gene expression level; the infection of Mφs led to a significant increase in TNF-α, IL-1β, and IL-6 mRNA levels, and IL-6 mRNA level was further enhanced with IFN-γ addition (Figure 6D, *p* < 0.01). Likewise, co-incubation with apigenin had no effect on *Tc* + IFN-γ induced cytokines’ gene expression in Mφs (Figure 6D).

In contrast, co-treatment with apigenin for 18 h resulted in 2- and 17-fold inhibition of the *Tc* + IFN-γ induced ROS (Figure 6E, *p* < 0.001) and NO (Figure 6F, *p* < 0.001), respectively; and 2- and 13-fold inhibition of the LPS + IFN-γ induced ROS (Figure 6G, *p* < 0.001) and NO (Figure 6H, *p* < 0.001), respectively, in RAW264.7 Mφs. The decline in *Tc* + IFN-γ induced ROS by apigenin co-treatment was confirmed in BMDMs (Figure 6I, *p* < 0.001). Co-treatment with another GLUT1 inhibitor, STF31, also abolished the ROS response (Figure 6J, *p* < 0.001) and diminished the NO response by 50% (Figure 6K, *p* < 0.001) in *Tc* + IFN-γ-induced Mφs. These results confirm our findings, and demonstrate that Mφ stimulation of pro-inflammatory cytokine response by *Tc* or LPS (+IFNγ) is not dependent on glucose metabolism, while glucose uptake is critical for ROS and NO production in infected Mφs.

### Glycolysis Supports the PPP in Pro-inflammatory Mφs

**Figure 7:**
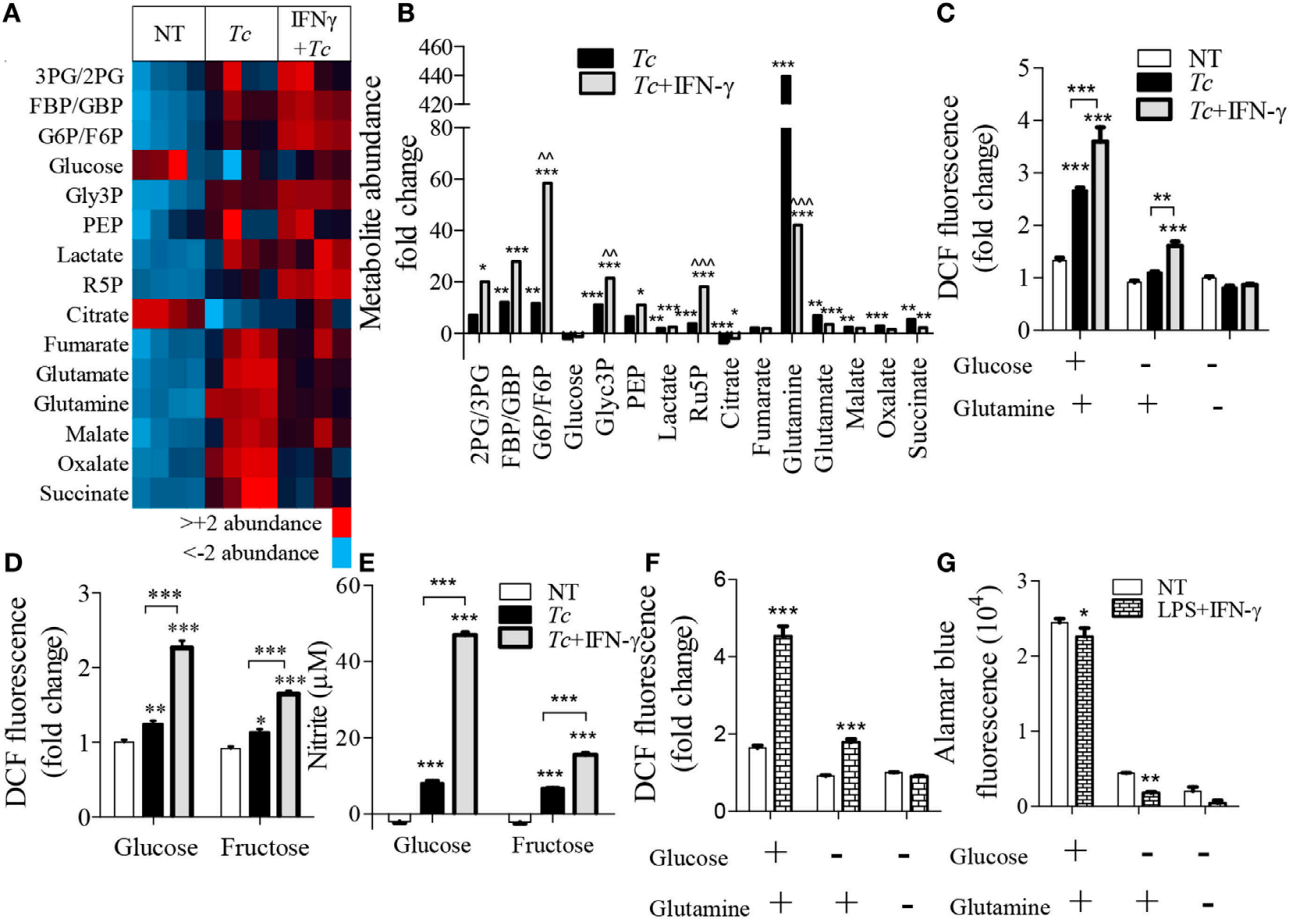
Relevance of glycolysis and pentose phosphate shunt in macrophage activation by *Trypanosoma cruzi* (*Tc*). RAW 264.7 macrophages were incubated for 18 h with *Tc* in presence or absence of IFN-γ **(A–E)** or with lipopolysaccharide (LPS) + IFN-γ **(F,G)**. **(A,B)** Targeted profiling of the glycolysis and Krebs cycle metabolites was performed by LC-MS, and data are presented as heat map **(A)** and fold change compared to no-treatment controls with false discovery rate of 0.25 **(B)**. **(C–E)** Macrophages were incubated for 18 h with *Tc* ± IFN-γ in the absence or presence of glucose, glutamine, or fructose. The intracellular reactive oxygen species (ROS) **(C,D)** and nitrite release **(E)** were measured by DCF fluorescence and Griess assay, respectively. **(F,G)** Macrophages were incubated for 18 h with LPS + IFN-γ in the presence or absence of glucose and glutamine. ROS **(F)** was determined by DCF fluorescence and cell viability **(G)** was analyzed by Alamar blue fluorescence. Data are shown as the mean of ≥3 biological replicates ± SEM. ANOVA_B–H_ for metabolite analyses and ANOVA_BF_ for ROS and nitric oxide (NO) levels were used, where *p < 0.05, **p < 0.01, and ***p < 0.001 are compared to no-treatment controls, ^^^^*p* < 0.01 compared to the *Tc*-treated group, otherwise indicated by a line over compared groups. Abbreviations: 3PG/2PG, 3-phosphogluconate/2-phosphogluconate; GBP/FBP, glucose 1,6-bisphosphate/fructose 1,6-bisphosphate; Glyc3P, glycerol 3-phosphate; PEP, phosphoenolpyruvate; Ru5P, ribulose 5-phosphate.

To delineate the steps in glycolysis and Krebs cycle that signal the differential ROS/NO response, we performed targeted metabolite profiling of the *Tc*-challenged Mφs (±IFN-γ). The detailed data are presented in Table S2 in Supplementary Material. We noted increased abundance of glycolysis [FBP/GBP, G6P/F6P, glycerol 3-phosphate (Glyc3P), lactate, and Ru5P] and Krebs cycle (fumarate, malate, oxalate, and succinate) metabolite intermediates in infected (vs. normal) Mφs (Figure 7A). Co-incubation with IFN-γ resulted in a major shift toward glycolysis, evidenced by >2-fold increase in the glycolysis metabolites [2PG/3PG, FBP/GBP, Glyc3P, phosphoenolpyruvate (PEP), and lactate] and ribulose-5-P from the PPP in infected Mφs (Figure 7B, all *p* < 0.001). Glucose and citrate levels declined in both of these groups, glutamine was particularly high (>10-fold, *p* < 0.001) in infected Mφs, and G6P/F6P, Glyc3P, and Ru5P were twofold to sixfold higher in *Tc* + IFN-γ (vs. *Tc*-infected) Mφs (Figure 7B, all *p* < 0.01).

To elucidate the essentiality of glycolysis linkage to PPP in ROS production, we stimulated Mφs with *Tc* (±IFN-γ) in the presence and absence of glutamine, glucose, or fructose for 18 h (Figures 7C–E). Glutamine can replenish the Krebs cycle by converting to glutamate and α-ketoglutarate. Glucose is used by both glycolysis and PPP, while fructose does not directly support the PPP as it enters the glycolysis pathway after the divergence to the pentose phosphate shunt. We found that glucose, but not glutamine, supplementation elicited a 2.5-fold increase in ROS response in infected Mφs, which was further intensified in *Tc* + IFN-γ stimulated Mφs (Figures 7C,D, *p* < 0.001). Incubation with fructose allowed <50% increase in the ROS level than was noted in glucose supplemented, *Tc* + IFN-γ stimulated Mφs (Figure 7D, *p* < 0.001). The NO release by Mφs responding to *Tc* + IFN-γ was also declined by threefold when glucose was replaced with fructose in the culture media (Figure 7E). Similar patterns of ROS production were noted in LPS + IFN-γ activated Mφs where the lack of glucose availability suppressed the ROS level (Figure 7F) and cell viability (Figure 7G). Together, the results presented in Figure 7 suggest that glycolysis shunt toward PPP supports the ROS/NO production in Mφs stimulated with *Tc* or LPS (+IFN-γ).

### Pentose Phosphate Shunt Is Essential for ROS/NO Response of Mφs against *Tc*

**Figure 8:**
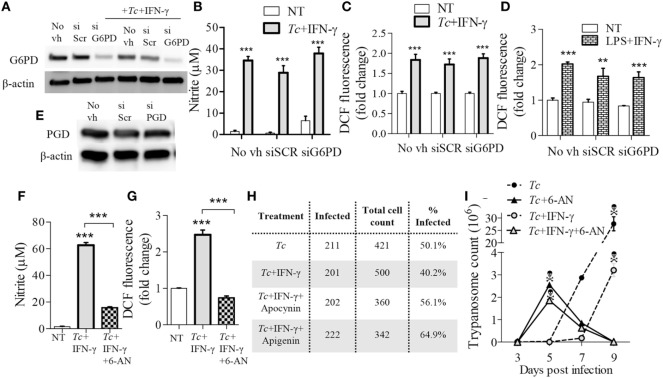
Reliance on pentose phosphate shunt by *Trypanosoma cruzi* (*Tc*) + IFN-γ-activated macrophages. **(A–E)** RAW 264.7 macrophages were transfected with glucose-6-phosphate dehydrogenase (G6PD), 6-Phosphogluconate dehydrogenase (PGD), or scrambled (SCR) siRNAs, or treated with media in absence of transfection vehicle (No vh) for 24 h. Transfected (and control) macrophages were then incubated for 18 h with *Tc* ± IFN-γ or lipopolysaccharide (LPS) + IFN-γ for 18 h. Shown are G6PD **(A)** and PGD **(E)** protein levels (β-actin, loading control) by western blotting, and nitric oxide (NO) release **(B)**, and intracellular reactive oxygen species (ROS) levels **(C,D)** by Griess assay and DCF fluorescence, respectively. **(F–I)** RAW 264.7 macrophages were incubated for 18 h with *Tc* in the presence or absence of IFN-γ (50 ng/mL), 6-aminonicotinamide (6-AN; 100 µM, inhibits PGD), apocynin (1 mM), or apigenin (100 µM). The NO release **(F)** and intracellular ROS **(G)** were measured as above. Macrophages were fixed at 18 h post-infection (pi), stained with Giemsa-Wright solution, and then ≥200 *Tc*-bearing macrophages were counted. The percentages of infected cells are reported in panel H. Trypanosome release from infected (±treatment) macrophages was quantified at 3, 5, 7, and 9 days pi **(I)**. Data are presented as the mean ± SEM of ≥3 biological replicates. ANOVA_Tukey_ **p < 0.01 and ***p < 0.001 in comparison to no-treatment controls unless indicated by a line over compared groups.

To validate the role of PPP in ROS/NO production, we applied an RNAi approach to inhibit the expression of glucose-6-phosphate dehydrogenase (G6PD) that is the first step linking glycolysis to PPP, and of 6-phosphogluconate dehydrogenase (PGD) that is the third enzyme in the PPP which catalyzes the production of ribulose-5-phosphate (Ru5P) and NADPH. The G6PD siRNA, but not the scrambled siRNA, resulted in 50% decline in G6PD expression (Figure 8A). Yet, G6PD inhibition by siRNA had no major effects on NO (Figure 8B) and ROS (Figure 8C) levels in *Tc* + IFN-γ stimulated Mφs. The LPS + IFN-γ stimulated Mφs also did not exhibit a decline in ROS production with G6PD knockdown (Figure 8D). Silencing of the PGD, even upon use of high concentrations of PGD siRNA duplex, could not be achieved (Figure 8E). Therefore, we resorted to using the pharmacological inhibitor of PGD, 6-aminonicotinamide (6-AN; 10 µM) during Mφ activation. Co-treatment with 6-AN abolished the NO (Figure 8F, *p* < 0.001) and ROS (Figure 8G, *p* < 0.001) formation in *Tc* + IFN-γ stimulated Mφs. These results confirm that glycolytic-PP shunt, and particularly the step producing Ru5P, is essential for ROS and NO response in Mφs infected by *T. cruzi*.

Finally, we determined the significance of glucose/PPP in controlling parasites in Mφs. Up to 50% of the RAW264.7 Mφs were infected with *Tc* (1:3 cell parasite ratio) at 18 h, and co-incubation with IFN-γ resulted in a 20% decline in the number of infected Mφs (Figure 8H). Inhibition of glucose transport (apigenin) or NOX2 (apocynin) abolished the IFN-γ effects in limiting parasites in Mφs. Further, treatment of Mφs with 6-AN (inhibits glucose-PPP link) enhanced parasite development in Mφs (Figure 8H). The peak release of trypomastigotes from *Tc*-infected (±IFN-γ) Mφs was observed during 7–9 days pi (Figure 8I). Co-incubation with 6-AN resulted in peak trypomastigotes release at 5 days pi after which cells began to die (Figure 8I). These results demonstrate that glucose-PPP support the NOX2/ROS production for early control of parasite replication in Mφs.

## Discussion

In this study, we investigated how the Mφ ability to control *Tc* infection is subdued, and took a metabolic approach to improve the trypanocidal function of Mφs. For this, we have used primary and cultured Mφs coupled with genetic and chemical depletion of PPARs, metabolite profiling followed by supplementation of critical metabolites and chemical and RNAi inhibition of key metabolic enzymes to delineate the Mφ response to *Tc*. Our data showed that Mφs are infected by *Tc* and elicit a potent, inflammatory cytokine response; however, they lack effective ROS and NO responses against the parasite. We showed that this is due to Mφs continuing to maintain a PPAR-α-dependent, Krebs cycle-linked oxidative metabolism that has no effect on inflammatory cytokine response, but allow only a partial level of NOX2/iNOS activation upon *Tc* infection. IFN-γ treatment dispensed the Mφs’ need to utilize pyruvate-supported Krebs cycle, led to complete metabolic shut down of oxidative metabolism, and enhanced the glycolytic source of energy availability in infected Mφs. Ironically, the glucose uptake and metabolism was diverted toward PPP, and the activity of PGD, which produces Ru5P and NADPH, was essential for eliciting potent ROS and NO response in infected Mφs. Inhibition of PGD led to decreased ROS/NO levels, increase in parasite replication, and earlier release of trypomastigotes from infected Mφs. To the best of our knowledge, this is the first study providing a detailed view of the metabolic regulation of the Mφ response to an intracellular pathogen, *T. cruzi*. We propose that chemical analogs enhancing the glucose–pentose phosphate shunt will be beneficial in controlling early parasite replication and dissemination in the infected host.

The TLR family of innate immune receptors (TLR1–TLR10) recognizes a variety of pathogen ligands and initiate two major pathways. The MyD88-dependent pathway is used by all TLRs except by TLR3 for the activation of NF-κB and activator protein -1 (AP-1) transcription factors, while the IFN-β-dependent pathway initiated by TLRs 3 and 4 activates the TIR-domain-containing adaptor protein and type I IFN responses. *T. cruzi and Tc* antigens (e.g., GPI-anchored mucins, cruzipain) are shown to engage TLR2 and TLR4, and *Tc* DNA is recognized by TLR9 to induce pro-inflammatory cytokine response in Mφs (22). Unfortunately, the *Tc* induced cytokine response seems insufficient in controlling parasites. Rather, the predominance of pro-inflammatory cytokines, IFN-γ, TNF-α, IL-17, with TLR2 and TLR4 involvement is pathological to the host, and suggested to be involved in the presentation of cardiac form of disease in infected humans (23). TLR2 and TLR4 are also suggested to be involved in the production of ROS and NO (24); however, in the context of *Tc* infection, TLR2/TLR4 activation appeared to be not sufficient to induce potent ROS/NO response to kill the intracellular parasite (25). Others have suggested that it is not the inability of Mφs to mount an ROS/NO response, rather the parasite’s elaborate antioxidant system of trypanothione-dependent tryparedoxin peroxidases dismutates the ROS/NO (12, 26). Our study suggests that this might not be the only cause for a sub-par ROS/NO response. Supplementing with IFN-γ during infection was sufficient to activate the mφ expression and activation of iNOS and NOX2, and production of NO and ROS, respectively. How ROS influences parasite control in Mφs have been controversial, including that low levels of supplied ROS or gp91phox knockout aids in parasite replication, while high levels of ROS are toxic to *T. cruzi* (27). Further, Paiva et al have suggested that oxidative stress fuels *T. cruzi* infection in mice (28); while studies from our laboratory indicate that inhibition of the antioxidant mechanisms resulted in increased tissue pathology in chagasic murine myocardium (29). These studies illuminate the complex fine gauging of ROS levels necessary for effective *Tc* killing by Mφs. Further studies will be required to identify how IFN-γ complements the *Tc*-generated stimuli in enhancing the ROS/NO response above the threshold for parasite killing. The two pathways that deserve immediate attention include the interaction of the atypical PKC ζ and TLR2 in the lipid rafts of the plasma membrane and the TLR4-MyD88-IL-1 receptor-associated kinase 4 signaling pathway that activate the p38 MAPK and protein kinase B pathways, which can initiate the phosphorylation of p47^phox^ and subsequent activation of NOX2 and ROS production in Mφs (30, 31).

How energy metabolism is reconfigured to support Mφ activation and effector function has not been fully studied. PPARs (α, γ, and δ isoforms) are ligand-activated transcription factors that regulate nearly every facet of fatty acid metabolism. Recent studies have established a role for PPAR-γ and -δ in the regulation of Mφ lipid metabolism and inflammation. Several groups have noted upregulation of PPAR-γ in murine and human Mφs, and initial studies suggested that PPAR-γ attenuates the pro-inflammatory Mφ response (19). Agonism of PPAR-γ coupled the uptake of oxidized low density lipoprotein to cholesterol efflux *via* induction of liver X receptor-α-mediated transcriptional cascade and the cholesterol efflux pump Abca1, and was beneficial in providing the atheroprotective effects in diabetes (32). Others have suggested that PPAR-δ-dependent shift toward oxidative metabolism is accompanied by an influx of fatty acids, and PPAR-δ-dependent surge in monounsaturated fatty acids synergize with IL-4 to enhance the alternative gene expression signature (33). Macrophage-specific knockdown of PPAR-γ resulted in a loss of alternatively activated Mφs in tissues and increased susceptibility to diet induced obesity, insulin resistance, and glucose intolerance in mice (34). The role of PPAR-α in Mφs is comparatively less studied. In a *T. cruzi* infection mouse model, PPAR-α mRNA expression has been shown to be enhanced in peritoneal Mφs isolated from mice at 6 days post-infection (20). Our study provides evidence that PPAR-α protein level also increases in murine Mφs by *Tc*-generated stimulus. Interestingly, genetic and chemical ablation of PPAR-α activity had no effect on the inflammatory cytokine response of infected Mφs; however, PPAR-α appeared to stimulate the ROS and NO production, at least partially, in response to initial infection. Inhibition of PPAR-α decreased the oxidative metabolism and the NO and ROS response in infected Mφs. A similar suppression of NO and ROS was observed with the inhibition of pyruvate transport to the mitochondria early in pro-inflammatory activation, which overall suggested that PPAR-α-regulated mitochondrial metabolism may be involved in the early events that lead to ROS and NO production by infected Mφs. Inhibiting mitochondrial pyruvate transport had been shown to suppress iNOS gene transcription in LPS-activated Mφs (35). A broken Krebs cycle results in accumulation of metabolites, e.g., succinate, which signaled transcriptional activation of inflammatory response in LPS-stimulated Mφs (21). We also observed a moderate increase in Krebs cycle metabolites including succinate in infected Mφs. Alternatively, a functional mitochondrial Krebs cycle could support the production of oxidative compounds as it has two enzymes that produce NADPH, which is utilized by NOX2 and iNOS for synthesizing ROS and NO, respectively. Further studies will be required to delineate the comparative role of PPAR-α in providing transcriptional and metabolic signals for regulating the NOX2/iNOS activation in Mφs. Regardless, PPAR-α inhibition was not sufficient to arrest parasite replication in Mφs.

Recent studies have described an increase in glycolysis-dependent lactate formation and activation of PPP in Mφs after phagocytosis (13), and that inhibition of sedoheptulose kinase (CARKL), which resulted in accumulation of metabolites in the PPP and a decline in Krebs cycle metabolites, enhanced the pro-inflammatory cytokine response in LPS-activated Mφs (36). Others showed that HIF1α-dependent transcriptional programming is responsible for heightening glycolysis in Mφs (14), and GLUT1-mediated glucose uptake drives a pro-inflammatory phenotype (37). In this study, we provide evidence that first few steps where glycolysis and PPP are linked, primarily support ROS and NO production in inflammatory Mφs. Our data show that in all instances when glucose availability was limited, achieved by inhibition of GLUT1, removal of glucose, or replacement of glucose with fructose as a carbon source, the levels of cytokine release were not largely altered while ROS and NO responses were abolished in *Tc-* and LPS-stimulated Mφs. IL-6 release were initially delayed with the inhibition of pyruvate transport or GLUT1, which may suggest an early transcription factor of IL-6 is influenced by metabolic signaling such as that demonstrated by cyclic adenosine monophosphate levels (38). Glutamine, which can replenish the Krebs cycle through its conversion to glutamate and α-ketoglutarate, was also insufficient in the absence of glucose to support cell viability and the ROS/NO response of activated Mφs. The increase in abundance of glucose-6-P/fructose-6-P and glycerol-3-P metabolites of glycolysis pathway and Ru5P of the PPP that reversibly fuel each other provided the first indication that activation of potent ROS/NO response in Mφs requires a coordinated regulation of glycolysis to PPP, instead of glycolytic formation of pyruvate or lactate. This was also evidenced by the findings that ROS/NO response was abolished by inhibition of PGD (produces Ru5P and NADPH) in infected Mφs. Future studies will be required to delineate if glycolytic-pentose phosphate shunt simply provides the NADPH substrate, or it provides metabolic signaling at transcriptional, translational, or posttranslational levels for NOX2 and iNOS activation in pro-inflammatory Mφs. Yet, we surmise that activation of glycolytic-pentose phosphate shunt will be beneficial in enhancing the Mφs’ ability to achieve early clearance of intracellular parasites.

In summary, the present study shows that early inhibition of mitochondrial metabolism may be detrimental for ROS and NO generation, glycolysis and PPP are important for ROS and NO generation at the metabolite level, and *Tc* infection may program the metabolism of host Mφs differentially at the pentose phosphate shunt compared to pro-inflammatory Mφs. Based on these findings, we extend our understanding of how the synthesis or release of ROS and NO compounds are controlled by metabolism, which may be further studied for potential translational purposes.

## Materials and Methods

### Ethics Statement

All bone marrow harvesting from mice were performed according to the National Institutes of Health Guide for Care and Use of Experimental Animals, and approved by the Institutional Animal Care and Use Committee at the University of Texas Medical Branch (protocol number: 0805029).

### *T. cruzi*, Cell Culture, and Treatment

*Trypanosoma cruzi* trypomastigotes Sylvio X10/4 isolate (ATCC, Manassas, VT, USA) were propagated in C2C12 murine skeletal muscle cells (ATCC) in RPMI medium (Corning, Corning, NY, USA) containing 5% FBS (GE Healthcare, Chicago, IL, USA) and penicillin/streptomycin (Corning).

RAW 264.7 murine Mφs were purchased from ATCC, and grown in DMEM medium containing 4.5 g/L glucose (Corning), 10% heat-inactivated FBS, and penicillin/streptomycin. RAW264.7 Mφs were used up to passage 14, and maintained in DMEM with 2% FBS for all experiments. Mycoplasma was tested negative by visualization of cells with DAPI stain and no amplification of mycoplasma genes shown by PCR and agarose gel electrophoresis.

The PPAR-α^−/−^ mice (B6:129S4-*Ppara^tm1Gonz^*/J) and matching WT mice were purchased from Jackson Laboratories (Bar Harbor, ME, USA). The bone marrow cells were isolated from the femurs of mice following standard protocol (39). Bone marrow cells were matured into Mφs over 9 days using 20 ng/mL macrophage-colony stimulating factor (M-CSF; Biolegend, San Diego, CA, USA) in DMEM and Ham’s F-12 50:50 mix culture medium (Corning). When used for experiments, BMDMs were maintained in 10% FBS/5-ng/mL M-CSF.

For all experiments, parasite: to cell ratio of 3:1 was used that provided infection of more than 50% of Mφs. When using lower parasite dose (1:1 ratio), cells do not become infected, while at higher parasite dose, cell death was observed before 18 h time point. In some experiments, Mφs were infected and incubated for 0, 3, 6, 12, and 18 h at the same time with or without 50 ng/mL of mouse recombinant interferon gamma (IFN-γ, Gibco, Carlsbad, CA, USA), PPAR-α agonist 50 µM WY14643 (Tocris Bioscience), or antagonist (5–50 µM GW6471, Tocris Bioscience, Minneapolis, MN, USA). GW6471 antagonist has been shown to bind to the ligand-binding domain of PPAR-α and the motif of the SMRT corepressor, preventing the active conformation of PPAR-α (40). NOX2 inhibitor (1 mM apocynin, Tocris Bioscience; or 10 µM DPI chloride, Sigma Aldrich, St. Louis, MO, USA), glucose transport inhibitor (100 µM apigenin, Cayman Chemical, Ann Arbor, MI, USA; or 100 µM SF-31, EMD Millipore, Billerica, MA, USA), pyruvate transport inhibitor (100 µM UK5099, Tocris Bioscience), and phosphogluconate dehydrogenase inhibitor (10 µM 6-AN Sigma-Aldrich). To evaluate the source of carbon needed for Mφs, cells were incubated in media that was free of glucose, glutamine, and pyruvate (Gibco) and supplemented with 24 mM d-glucose (Fluka, St. Louis, MO, USA), 1 mM pyruvate, 24 mM d-fructose (Fisher Scientific, Waltham, MA, USA), or 10 mM glutamine (Atlanta Biologicals, Flowery Branch, GA, USA). Mφs classically activated with 100 ng/mL LPS (Sigma-Aldrich) and 20 ng/mL IFN-γ were used as positive controls. All chemicals were of >99% purity, and of molecular and cell biology grade.

### Cell Lysis, Fractionation, and Western Blotting

To prepare total cell lysates, Mφs (1–2 × 10^6^) were washed twice with PBS, and lysed in 200 µL of RIPA buffer containing 150 mM NaCl, 1% Triton X-100, 0.5% sodium deoxycholate, 0.1% SDS, and 50 mM Tris at pH 8.0. The samples were sonicated on ice, twice for 10 s each, and centrifuged at 10,000 rpm for 10 min at 4°C to collect the membrane-cleared supernatants.

To prepare nuclear fractions, Mφs (1 × 10^7^) were washed as above, and isolated using the sucrose buffer method as previously described (41). All cell lysates and fractions were stored at −80°C until analysis, and protein concentration in all samples was quantified by using the bicinchoninic acid assay (Thermo Scientific) by following manufacturer’s instructions.

For western blotting, samples (20 µg) were resolved on 10% polyacrylamide gels (Sigma-Aldrich) at 100 V for 90 min, and proteins were wet-transferred to PVDF membranes (EMD Millipore). The membranes were blocked for 1 h with 3% BSA (Fisher Scientific), and then incubated overnight at 4°C with antibodies against PPAR-α (H-2), PPAR-β (F-10), PPAR-γ (E-8), or gp91phox (NL-7). All four antibodies were from Santa Cruz Biotechnology (Dallas, TX, USA) and used at 1:500 dilutions in 3% BSA. The membranes were washed three times with TBST (TBS + 0.1% Tween-20; Fisher Scientific), and incubated for 1 h at room temperature with HRP-conjugated anti-mouse antibody (Southern Biotech, Birmingham, AL, USA, 1: 10,000 dilution in 3% BSA). Proteins with antibody conjugation were detected by using the chemiluminescence based method (GE Healthcare, Chicago, IL, USA), and imaged and analyzed by using an Image Quant LAS4000 system (GE Healthcare) (42). Nuclear proteins were normalized against TATA binding protein (Abcam, Cambridge, MA, USA, 1:1,000 dilution in 3% BSA) and proteins from total cell lysates were normalized against β-actin (Santa Cruz Biotechnology).

### Cell Lysis and Enzyme-Linked Immunosorbant Assay (ELISA)

Cytokines released by Mφs were measured in the culture media by using IL-6, IL-1β, and TNF-α ELISA by following recommended instructions (BD Biosciences, San Jose, CA, USA). The change in absorbance as a measure of cytokine concentration was monitored by using a SpectraMax M5 spectrophotometer (Molecular Devices, Sunnyvale, CA, USA).

For the measurement of intracellular cytokines, 1 × 10^6^ macrophages were washed and re-suspended in 500 µL PBS then sonicated on ice. Lysate was centrifuged at 10,000 rpm for 10 min at 4°C for the collection of membrane-cleared supernatants.

### Functional Metabolic Status of Mφs

The indices of mitochondrial respiration and glycolysis were analyzed in RAW264.7 Mφs (±treatment) by using a Seahorse XF24 Extracellular Flux Analyzer (Agilent, Santa Clara, CA, USA) conducted by Dr. Bartosz Szczesny in the group of Dr. Csaba Szabo, as previously described (5). Each treatment was performed in quadruplicates, and analyzed in triplicates. In brief, 80,000 Mφs were incubated for 3 or 18 h with media only, *Tc, Tc* + IFN-γ, GW6471 only, or *Tc* + GW6471. Cells were then equilibrated in assay medium, and OCR (moles per minute) was measured as an index of mitochondrial function. After recording the basal OCR, OCR was recorded with sequential addition of 1 µM each of oligomycin (inhibits complex V), FCCP (uncouples respiration and ATP synthesis), and antimycin A (inhibits complex III). Data were used to calculate the ATP production rate (OCR_basal_ − OCR_oligomycin_) as previously shown in Ref. (5).

The indices of glycolytic function were monitored by the ECAR (mpH per minute) based on lactate efflux, and were determined simultaneously with the OCR as previously reported (5). The ECAR at the basal level (ECAR_Basal_) represented the use of glycolysis for energy demand. The total glycolytic capacity was calculated as the difference between ECAR_Basal_ and ECAR with oligomycin addition. OCR and ECAR were normalized by per microgram of total protein.

### Measurement of ROS and NO

To measure intracellular ROS, 50 µM of the cell-permeant 2′,7′ dichlorodihydrofluorescein diacetate (H_2_DCFDA, Molecular Probes, Eugene, OR, USA) was loaded onto cells in clear bottom, black walled plates (Fisher Scientific). Cells were incubated for 30 min, washed twice with PBS, and then loaded with 100 µL PBS. Fluorescence of oxidized product (2′,7′-dichlorofluorescein, DCF) was measured at excitation/emission of 493/520 nm using the Spectramax M5 Fluorimeter (Molecular Devices).

The NO level was measured by assaying the concentrations of nitrite (a stable NO breakdown product) in the culture supernatants by using the Griess assay as previously described (43).

### Gene Expression Analysis by Reverse Transcription Quantitative PCR

Mφs (1 × 10^6^ ± *Tc* ± IFN-γ) were washed with PBS, and total RNA was extracted by using the Aurum Total RNA mini kit (Bio-Rad, Hercules, CA, USA) or TRIzol Reagent (Invitrogen, Carlsbad, CA, USA) and re-suspended in 30 µL of TE buffer. RNA was reverse transcribed then qPCR using SYBR Green (Bio-Rad, Hercules, CA, USA) and 3 µM of gene-specific oligonucleotide pairs (Table S1 in Supplementary Material) were performed as previously described (5). The threshold cycle (*C_T_*) values for target mRNAs were normalized to the *C_T_* values for the HPRT housekeeping gene sequence (ΔC*_T_*), and then converted to linear scale as 2^(ΔCT)^. The relative fold changes in gene expression were calculated compared to no-treatment controls.

### Targeted Profiling of Metabolites

*Reagents and internal standards*: High-performance liquid chromatography (HPLC) grade acetonitrile, methanol, and water were purchased from Burdick & Jackson (Morristown, NJ, USA). Mass spectrometry grade formic acid and the internal standards, Tryptophan-15N2, Glutamic acid-d5, Thymine-d4, Gibberellic acid, Trans-Zeatin, Jasmonic acid, Anthranilic acid, and Testosterone-d3 were purchased from Sigma-Aldrich. The calibration solution containing multiple calibrants in acetonitrile/trifluroacetic acid/water was purchased from Agilent Technologies.

*Cell treatments*: RAW264.7 Mφs (5 × 10^6^) were seeded in T25 flasks, and incubated with media alone, *Tc*, or *Tc* + IFN-γ for 18 h. Cell samples (quadruplicates per treatment) were harvested by using the non-enzymatic cell dissociation solution (C5914, Sigma-Aldrich). Cells were washed twice with PBS, counted by using a hemocytometer, pelleted, snap frozen in liquid nitrogen, and then stored at −80°C. Targeted measurement of Krebs cycle and glycolysis metabolites was performed at the Baylor College of Medicine Metabolomics Core.

*Extraction of metabolites*: Cell pellets were taken through three cycles of freeze-thaw in liquid nitrogen and over ice, and then spiked with 750 µL of ice cold methanol: water (4:1) containing 0.05 mM of internal standards. Cells were homogenized, extracted by using a chloroform–methanol method, and examined by Single Reaction Monitoring as previously performed (44). For quality assurance, we co-analyzed the sample metabolites with four liver samples and normalized to two methods using the internal standards. Reproducibility of the metabolite profiling was determined by measuring the instrument variation and process variation, and liver samples spiked with internal standards were used to assess the quality of metabolite extraction. Metabolites were separated by normal phase chromatography by using methods as previously described (45).

*Liquid Chromatography-Mass spectrometry HPLC analysis* was performed using an Agilent 1290 series HPLC system equipped with a degasser, binary pump, thermostatted autosampler, and column oven (all from Agilent Technologies). The Multiple Reaction Monitoring-based measurement of relative metabolite levels, used either reverse phase or normal phase chromatographic separation. All samples were kept at 4°C and 5 µL was used for analysis.

*Identification of targeted metabolites*: The normal phase chromatographic separation was also used for targeted identification of metabolites by using the Krebs cycle protocol previously described (45), except that the flow rate was increased during the separation from 0.2 mL/min (0–20 min), 0.3 mL/min (20.1–25 min), 0.35 mL/min (25–30 min), 0.4 mL/min (30–37.99 min) and finally set at 0.2 mL/min (5 min). Metabolites were separated on a Luna Amino (NH2) column (4 µm, 100A 2.1 mm × 150 mm, Phenominex). Each sample was assessed in both positive and negative ionization modes by using a dual Electrospray Ionization, and the data were acquired by using a Mass Hunter Software (Agilent). The data were analyzed at 0.25 false discovery rate by *t*-test and Benjamini–Hochberg test for statistical significance between experimental groups.

### Cell Viability

For the analysis of cell viability, 1 × 10^5^ RAW264.7 Mφs were activated with LPS + IFN-γ for 17 h. Alamar Blue (Thermo Fisher) was added to cells in 96-well plate for 1 h before measuring fluorescence at Excitation/Emission of 560/590 nm.

### Visualization and Counting of *T. cruzi* Infection in Mφs

RAW 264.7 Mφs (5 × 10^4^ cells per well) were seeded in eight-chamber slides (Thermo Scientific), and infected or treated in triplicate. Cells were fixed for 5 min in methanol, and then flooded in Giemsa-Wright stain (Fisher Scientific) for 2 min. The cells were washed three times in pH 6.4 PBS buffer (Corning), air dried, mounted with Permount (Fisher Scientific) and coverslip, and visualized at 40× magnification on a light microscope (BX53F Olympus, Center Valley, PA, USA).

For counting of trypanosome release from Mφs, 5 × 10^5^ RAW 264.7 Mφs were seeded in 12-well plates, and infected with *Tc* at cell to parasite ratio of 1:3 in the presence or absence of IFN-γ and 6-AN in 5% FBS DMEM. After 24 h, Mφs were washed twice with PBS to remove free parasites, and then incubated in fresh media, IFN-γ and 6-AN for 48 h. Culture media was collected and replaced with fresh media only. The collected media were centrifuged at 1,800 × *g* for 10 min, and the trypanosome pellets were re-suspended in 15 µL of media before counting by light microscopy.

### Small Interfering RNA Transfection of RAW264.7 Mφs

Silencing of genes encoding for G6PD and PGD was performed by using specific siRNA duplexes directed against mouse G6PD (s66341, Thermo Scientific, 20 nM) and mouse PGD (110208 locus, OriGene, Rockville, MD, USA, 70 nM). RAW264.7 Mφs (5 × 10^5^ per well) were seeded to obtain 50% confluency and transfected with siRNA by using TKO transfection reagent (Mirus Bio, Madison, WI, USA) following manufacturer’s recommendations. After 24 h, Mφs were washed with PBS and used for various experiments.

### Statistical Analysis

All data were analyzed by using a Prism5 (Graphpad, San Diego, CA, USA) or SPSS (IBM, Chicago, IL, USA) software. Data are presented as the mean value of minimum of triplicate observations in two independent experiments ± the SEM, unless indicated. Significance was calculated by using one variable comparison analyses (one-way ANOVA) with Tukey’s *post hoc* test, and multiple comparison analyses (two-way ANOVA) with Bonferroni *post hoc* test. Significant differences compared to no-treatment controls or as otherwise stated are annotated as follows: *p value < 0.05; **p value < 0.01; and ***p value < 0.001.

## Author Contributions

Conceptualization: SJK and NJG; methodology: SJK and NJG; investigation: SJK, BS, and NP; resources: BS, XW, NP, and NJG; writing: SJK and NJG; review and editing:, SJK, BS, XW, NP, and NJG; supervision: NJG; funding acquisition: NJG.

## Conflict of Interest Statement

The authors declare that the research was conducted in the absence of any commercial or financial relationships that could be construed as a potential conflict of interest.

## Abbreviations

6-AN, 6-aminonicotinamide; BMDM, bone marrow-derived macrophage; cAMP, cyclic adenosine monophosphate; DPI, diphenyliodinium; ECAR, extracellular acidification rate; G6PD, glucose-6-phosphate dehydrogenase; GLUT1, glucose transporter 1; GM-CSF, granulocyte macrophage-colony stimulating factor; GPI, glycosylphosphatidylinositol; IL, interleukin; iNOS, inducible nitric oxide synthase; MyD88, myeloid differentiation primary-response protein 88; NF-κB, nuclear factor-κB; NO, nitric oxide; NOX2, NADPH oxidase 2; ${\text{O}}_{2}^{-}$, superoxide; OCR, oxygen consumption rate; PGD, 6-phosphogluconate dehydrogenase; pi, post-infection; PPAR, peroxisome proliferator-activated receptor; PPP, pentose phosphate pathway; Ru5P, ribulose-5-phosphate; ROS, reactive oxygen species; *Tc* or *T. cruzi, Trypanosoma cruzi*; TLR, toll-like receptor.
