# Supplementary material for: Pentose Phosphate Shunt Modulates Reactive Oxygen Species and Nitric Oxide Production Controlling Trypanosoma cruzi in Macrophages
Source: Front Immunol. 2018 Feb 16;9:202. doi: 10.3389/fimmu.2018.00202 (PMC5820298; doi:10.3389/fimmu.2018.00202)
Supplement: Supplementary file 1 [file Table_1.DOCX]

**S1 Table: Oligonucleotides used for RT-qPCR**

| Gene | NCBI Acc. # and oligonucleotide reference | Forward primer | Reverse primer | Amplicon size (bp) |
| --- | --- | --- | --- | --- |
| IL-1β | NC_000068.7 (28) | gagcttcaggcaggcag | gggatccacactctccagc | 439 |
| IL-6 | NM_031168.2 | ttctcatttccacgatttcccag | ttccatccagttgccttcttgg | 175 |
| TNF-α | NC_000083.6 (28) | gttctatggcccagaccctcaca | taccagggtttgagctcagc | 314 |
| HPRT | NM_013556.2 | cattgtggccctctgtgt | ctacagtcataggaatggatctatca | 110 |

All oligonucleotides were designed by using Primer-BLAST (33) based on sequences in corresponding National Center for Biotechnology Information (NCBI) accession numbers, and synthesized by IDT (San Jose CA).
