# Supplementary material for: Pentose Phosphate Shunt Modulates Reactive Oxygen Species and Nitric Oxide Production Controlling Trypanosoma cruzi in Macrophages
Source: Front Immunol. 2018 Feb 16;9:202. doi: 10.3389/fimmu.2018.00202 (PMC5820298; doi:10.3389/fimmu.2018.00202)
Supplement: Supplementary file 2 [file Table_2.DOCX]

**S2 Table. Abundance of glycolysis and Krebs cycle metabolites in mφs infected with *T. cruzi* (± IFN-γ).**

| **Metabolic**  **Pathway** | **Metabolites** | ***Tc*+IFN-γ vs NT** | | | ***Tc* vs NT** | | | ***Tc* vs *Tc*+IFN-γ** | | |
| --- | --- | --- | --- | --- | --- | --- | --- | --- | --- | --- |
|  |  | **Mean fold change** | p-value | | **Mean fold change** | p-value | | **Mean fold change** | p-value | |
|  |  |  | **t test** | **BH test** |  | **t test** | **BH test** |  | **t test** | **BH test** |
| Glycolysis | **3PG/2PG** | 20.09 | 0.008 | 0.013 | 7.09 | ns | ns | -2.83 | ns | ns |
|  | **GBP/FBP** | 27.92 | 0.000 | 0.000 | 12.15 | 0.001 | 0.002 | -2.30 | 0.049 | ns |
|  | **G6P/F6P** | 58.42 | 0.000 | 0.000 | 11.64 | 0.001 | 0.002 | -5.02 | 0.001 | 0.003 |
|  | **Glucose** | -1.35 | ns | ns | -2.12 | ns | ns | -1.57 | ns | ns |
|  | **Glyc3P** | 21.48 | 0.000 | 0.000 | 11.11 | 0.000 | 0.000 | -1.93 | 0.000 | 0.003 |
|  | **PEP** | 11.07 | 0.031 | 0.034 | 6.59 | ns | ns | -1.68 | ns | ns |
|  | **Lactate** | 2.45 | 0.000 | 0.000 | 2.06 | 0.004 | 0.004 | -1.19 | ns | ns |
|  | **Ru5P** | 18.08 | 0.000 | 0.000 | 3.84 | 0.000 | 0.000 | -4.71 | 0.000 | 0.000 |
| Krebs cycle | **Citrate** | -2.00 | 0.019 | 0.023 | -3.86 | 0.002 | 0.003 | -1.93 | ns | ns |
|  | **Fumarate** | 1.94 | 0.001 | 0.003 | 2.16 | 0.002 | 0.003 | 1.12 | ns | ns |
|  | **Glutamine** | 42.17 | 0.000 | 0.000 | 439.40 | 0.000 | 0.000 | 10.42 | 0.000 | 0.000 |
|  | **Glutamate** | 3.39 | 0.000 | 0.000 | 6.84 | 0.001 | 0.002 | 2.02 | ns | ns |
|  | **Malate** | 1.99 | 0.001 | 0.002 | 2.29 | 0.002 | 0.003 | 1.15 | ns | ns |
|  | **Oxalate** | 1.50 | 0.009 | 0.013 | 2.97 | 0.000 | 0.000 | 1.98 | 0.001 | 0.004 |
|  | **Succinate** | 2.18 | 0.003 | 0.005 | 5.42 | 0.001 | 0.002 | 2.49 | 0.028 | ns |

RAW 264.7 mφs were incubated with *Tc*, *Tc*+IFN-γ for 18 h. Cells not treated (NT), and incubated in media alone were used as controls. Glycolysis and Krebs cycle metabolite abundance in 5 x 10^6^ mφs were measured by LC-MS, and fold change values were calculated as described in Materials and Methods. Statistical significance was calculated with consideration of 0.25 false discovery rate. Mean fold changes at >2-fold level with p<0.05 in both t-test and Benjamini-Hochberg (B-H) test are underlined. ns: no significance, p>0.05. Abbreviations: 3PG/2PG - 3-phosphogluconate/2-phosphogluconate; GBP/FBP – Glucose 1,6-bisphosphate/Fructose 1,6-bisphosphate; Glyc3P – Glycerol 3-phosphate; PEP – Phosphoenolpyruvate; Ru5P – Ribulose 5-phosphate.
