# Supplementary figures and images for: Pentose Phosphate Shunt Modulates Reactive Oxygen Species and Nitric Oxide Production Controlling Trypanosoma cruzi in Macrophages
Source: Front Immunol. 2018 Feb 16;9:202. doi: 10.3389/fimmu.2018.00202 (PMC5820298; doi:10.3389/fimmu.2018.00202)

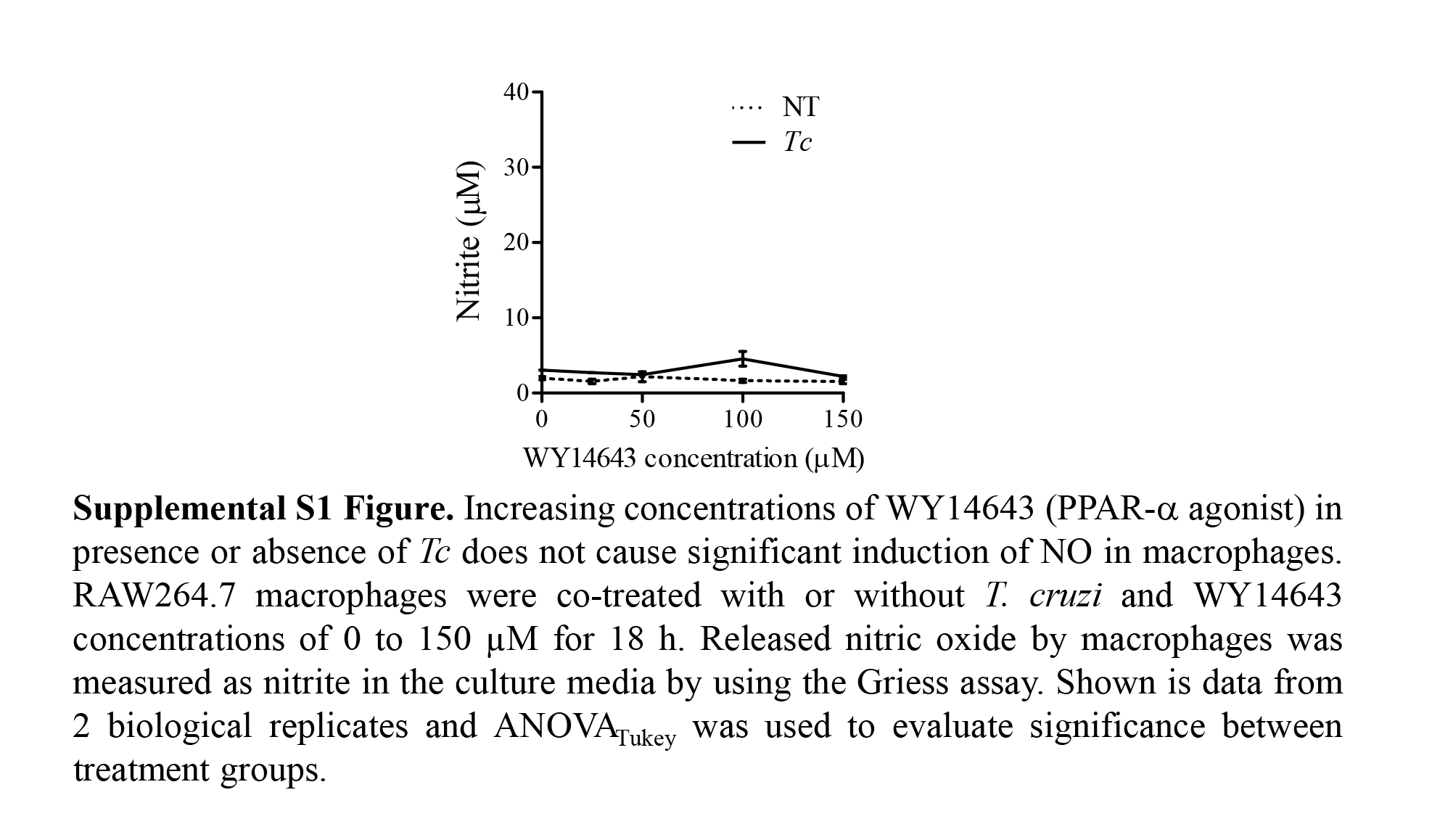

Supplement: Supplementary file 3 [file Image_1.TIF]

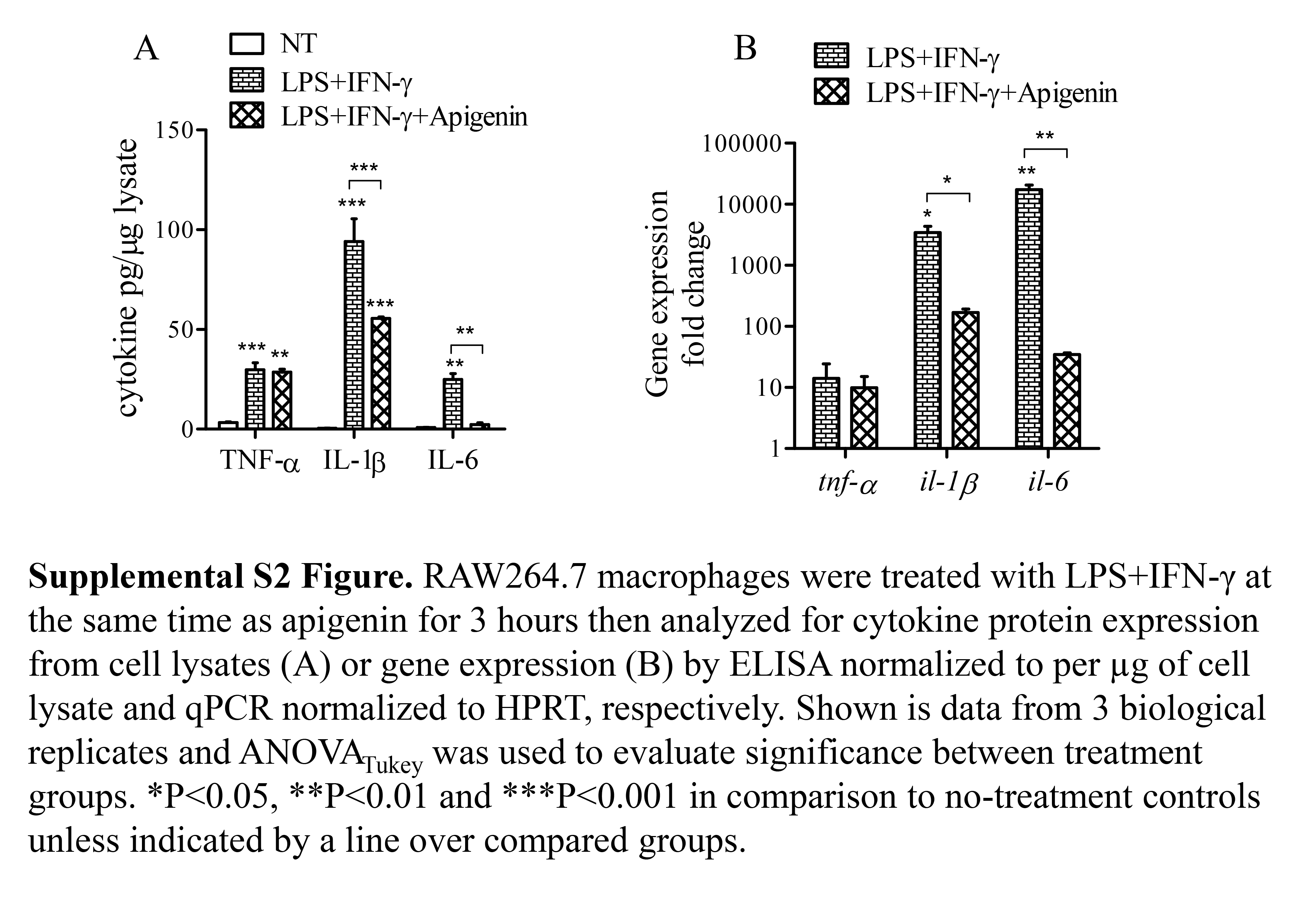

Supplement: Supplementary file 4 [file Image_2.TIF]
